# Cross-sectional case-control study on medical students’ psychosocial stress during COVID-19 pandemic in Hong Kong

**DOI:** 10.1016/j.heliyon.2021.e08486

**Published:** 2021-11-26

**Authors:** Michael Co, Margaret Kay Ho, Alina Ashok Bharwani, Vernice Hui Yan Chan, Evelyn Hui Yi Chan, Kam Sheung Poon

**Affiliations:** a Center for Education and Training, Department of Surgery, University of Hong Kong, Hong Kong; b Queen Elizabeth Hospital, Hospital Authority, Hong Kong

**Keywords:** Medical education, Psychosocial, COVID-19

## Abstract

**Introduction:**

COVID-19 pandemic has resulted in significant changes in pedagogy for undergraduate medical curriculum. Many physical clinical teachings have been replaced by online pedagogy. This study aims to evaluate the relation between medical students’ stress during COVID-19 pandemic and their academic performance at the final examination.

**Methods:**

This is a cross-sectional questionnaire-based study. Student's stress level were evaluated by the COVID-19 Student Stress Questionnaire (CSSQ). Correlation of stress level and students' performance at the final examination was performed.

**Results:**

110 out of 221 (49.8%) final-year medical students responded to the questionnaire, 13 students failed in the final examination (case) while 97 students passed in the final MBBS examination (control).

Baseline demographic data between case and control were comparable. The median age for both cases and controls were 24 years.

Compared to controls, cases reported higher levels of stress in all domains, namely in relation to risk of contagion, social isolation, interpersonal relationships with relatives, university colleagues and professors, academic life, and sexual life. Notably, a significantly higher proportion of cases reported academic-related stress compared to controls (p < 0.01), with 100% of cases perceiving their academic studying experience during the COVID-19 pandemic to be “very” or “extremely” stressful, compared to 35.1% of controls.

**Conclusion:**

Increased stress to academic and study during COVID-19 was associated with worse examination outcome at the final examination. Extra academic support will be needed to cater students’ need during the pandemic.

## 1 Introduction

The Coronavirus Disease 2019 (COVID-19) pandemic and the resultant social distancing measures implemented globally have had drastic impacts on medical education internationally. Social distancing and infection control regulations have forced medical institutions to modify their teaching strategies, with some even converting to online teaching entirely [1]. The “COVID-19 generation” of medical students have had to adapt from a primarily practice-based education format to distance learning, and as a result, may have missed out on educational experiences that were previously considered crucial [2]. This significant burden on medical students’ psychological well-being and stress may be further linked to their academic performance.

Numerous studies have reported on the negative mental health impacts of these rapid changes in medical education on medical students internationally; these impacts range from increased self-reported stress to clinically significant psychiatric illness. One study conducted on medical students in the United Kingdom (UK) using a self-perceived stress scale found that 54.5% of participants had moderate to extreme stress levels. In another study conducted across 3 medical schools in Western China, 82.3% of the 361 medical students surveyed had moderate to high levels of reported stress [3].

Stress in medical students during COVID-19 is correlated with a variety of risk factors, including those related to the fear of contagion, isolation, and interpersonal relationships. Health-related fears and social isolation were found in studies to contribute to psychosocial burden for medical students. Medical students’ proximity to teaching hospitals as a potential transmission source of infection for themselves or those they are living with may add to their stress [4]. This was echoed in a British study where stress levels of medical students were associated with concerns for personal health and the health of family members [5]. Social isolation due to social distancing policies and reduced in-person teaching was also found in studies to contribute to psychosocial burden across medical students. One study conducted in Saudi Arabia found that medical students had feelings of disheartenment and emotional detachment from family and friends during quarantine, which resulted in decreased study performance [6]. Another study reported a positive correlation between perceived mental stress and loneliness in Chinese medical students [7].

Additionally, stress related to COVID-19 may be linked to students' academic performance, given the abrupt changes in pedagogical methods adopted during this period. O'Byrne et al. reported that stress levels among UK medical students were significantly associated with transition to online learning and assessment formats [5]. Furthermore, a study in Hong Kong found medical students' performance in distance learning problem-based learning tutorials to be significantly lower than those in conventional face-to-face tutorials [8]. However, Syed et al. concluded that there was no significant difference in medical students' academic performance on a final exam for a first-year module during the pandemic [9].

This study aims to evaluate the psychosocial stressors and stress levels of final year undergraduate medical students from a Medical School in Hong Kong during COVID-19 in which teaching was conducted online. While previous studies demonstrated that online teaching during COVID-19 is technically feasible and produces comparable outcomes to face-to-face teaching with regards to HKU medical students’ performance and skills [10], it is uncertain whether the psychosocial wellbeing of students was impacted by the pandemic and the resulting adapted curriculum.

## 2 Methods

This is a prospective case control study. Study power is considered adequate when sample size is > 109. Sample size calculation was performed with two-sided confidence level (1-alpha) of 95% and a Power of 80%, assuming failure rate of 9–10%. Online questionnaire was distributed to all final year medical students by email on 1st July 2021 (i.e. 4 weeks after final examination to minimize recall bias). Questionnaire collection was opened until 110 responses were collected.

Medical students of Year of 2021 received final year medical curriculum during COVID-19 pandemic where strict social distancing measures were implemented. Students were not allowed to enter hospital premises during the outbreaks. Lectures and classes were conducted online unless specially arranged in small groups outside hospital premises (such as skills learning sessions in a group of 8–10 students).

**Figure 1:**
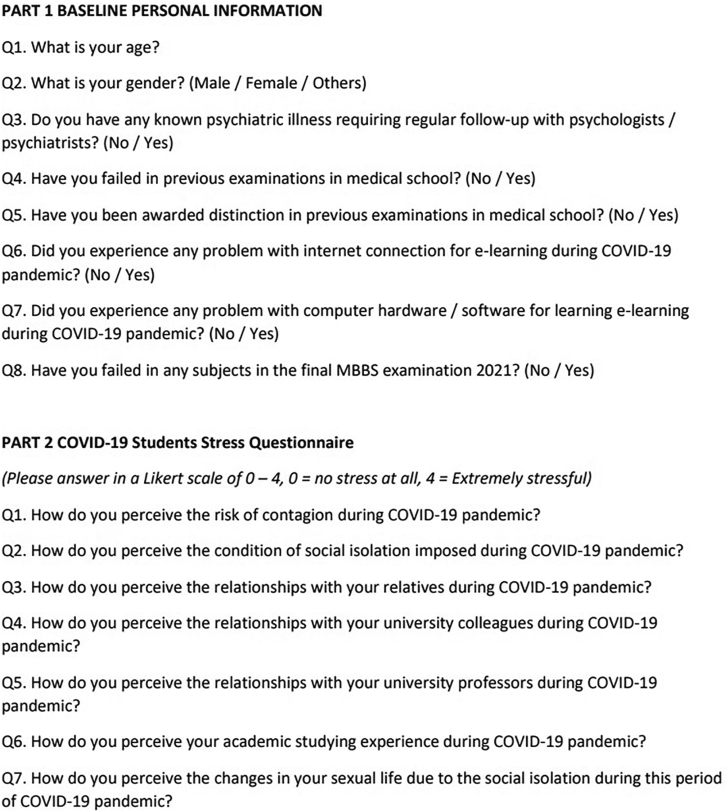
Questionnarie used in the study.

The online questionnaire contains two sections. Section 1 focused on baseline demographic data including age, gender, any history of psychiatric illness and previous academic performance. Section 2 focused on medical students’ stress response during COVID-19 pandemic using the validated COVID-19 Student Stress Questionnaire (CSSQ) developed by Zurlo, et al [11]. CSSQ consists of 7 questions (Figure 1). Time spent on the questionnaires were not compensated.

This is a case-control study. Students who failed in the final medical school examination were defined as Case group, while those who passed the final medical school examination were classified as Control group. Correlation between CSSQ stress response rating and examination performance was analysed. Categorical data were analysed with Chi-square test or Fishers' exact test, continuous data were analysed with Student's T-test.

## 3 Results

Online questionnaire was opened until 110 responses were collected. It took 3 weeks for 110 responses to be collected. 110 out of 221 (49.8%) final-year medical students responded to the questionnaire, 13 students failed in the final examination (case) while 97 students passed in the final MBBS examination (control). Median age was 24 (Range 23–27). 56 were male students and 54 were female students. 6 (5.5%) students had known psychiatric illness (depression, anxiety disorder) requiring psychiatry follow-up. 16 (14.5%) students had failed in previous examinations in the medical school. 11 (10%) students had been awarded distinction in previous examinations in the medical school. 10 (9.1%) students reported difficulty with internet connection for online learning, while 7 (6.4%) students reported difficulty with computer hardware/software for online learning.

Baseline demographic data between case and control were comparable. The median age for both cases and controls were 24 years (range: 24–26 years in cases, 23–27 years in controls). There were no significant differences between the two groups in terms of gender distribution, psychiatric illness history, failure or distinction in previous exams, or problems with internet connection and computer hardware/software. The baseline characteristics of each group were summarised in Table 1.

**Table 1:** Baseline demographics between the two groups.

**Factor**	**Case N = 13**	**Control N = 97**	**P-value**
Median age	24 (Range 24–26)	24 (Range 23–27)	1.0000
Gender	Male = 9	Male = 47	0.2391
Psychiatric illness history	1	5	0.5386
Failed in previous exams	4	12	0.4721
Distinction in previous exams	0	11	0.3357
Internet connection problem	1	9	1.0000
Computer hardware/software problem	1	6	1.0000

Compared to control group, students from case group reported higher levels of stress in all domains, namely in relation to risk of contagion, social isolation, interpersonal relationships with relatives, university colleagues and professors, academic life, and sexual life (Table 2). Notably, a significantly higher proportion of cases reported academic-related stress compared to controls (p < 0.01), with 100% of cases perceiving their academic studying experience during the COVID-19 pandemic to be “very” or “extremely” stressful, compared to 35.1% of controls.

**Table 2:** Number of students who reported “Very” or “Extremely” stressful in each domain.

**Question**	**Case N = 13**	**Control N = 97**	**P-value**
Q1 (COVID-19 infection)	2 (15.4%)	3 (3.1%)	0.1054
Q2 (Social isolation)	2 (15.4%)	5 (5.1%)	0.1926
Q3 (Relationship with family)	1 (7.7%)	1 (1.0%)	0.2234
Q4 (Relationship with peers)	3 (23.1%)	7 (7.2%)	0.0953
Q5 (Relationship with teachers)	2 (15.4%)	3 (3.1%)	0.1054
Q6 (Stress from academic work)	13 (100%)	34 (35.1%)	<0.0001
Q7 (Sexual relationship)	2 (15.4%)	6 (6.2%)	0.2396

Other variables which contributed to stress among both cases and controls include (in descending order of prevalence) relationship with peers (9.1%), sexual relationships (7.3%), social isolation (6.4%), risk of COVID-19 infection and relationship with teachers (equal prevalence of 4.5%), and relationship with family (1.8%). There was no significant difference found between cases and controls with regards to these variables.

## 4 Discussion

Academic stress during COVID-19 pandemic is associated with worse academic outcome as reflected by the poorer final examination performance among those who reported higher acdemic stress. Conversely, several stressors that were expected to negatively impact academic outcomes were ultimately not found to have had a significant impact in this study population. While multiple studies have explored the impact of COVID-19 on the psychological wellbeing of medical students, their experiences of and perceived stressors during the pandemic may vary widely depending on the sociocultural background. This study provides new insights into the specific experience of medical students in Hong Kong to contribute to the diverse body of literature on the unprecedented impacts of the COVID-19 pandemic on medical education and medical students.

Stress from academic work during COVID-19 was significantly higher in students who failed the final exam, compared to those who passed all subjects. Sources of academic stress may include various modifications made to medical education and pedagogy in response to the pandemic. The heightened risk of coming into contact with COVID-19 patients while attending teaching sessions conducted in clinical areas has been linked to increased anxiety-related issues [12]. Alternatively, in contexts where the mode of teaching was converted to online, stress levels were positively correlated with the transition to online learning and online assessment formats [5]. Additionally, a study on Chinese medical students engaged in online learning during COVID-19 reported stress levels to be the highest for academic-related stressors compared to psychosocial and health-related stressors, especially regarding “performance in examinations” [3].

Increased stress has been demonstrated to be detrimental to academic performance. Kötter et al. found that higher levels of academic stress among undergraduate medical students was predictive of poorer academic performance [13]. Similarly, Melaku et al. investigated the relationship between stress and academic performance in Ethiopian medical students and concluded that stress had a significant negative correlation with academic performance [14]. Another study from Lahore, Pakistan also found this relationship to be significant [15]. However, this was disputed by Abdulghani et al. who noted no significant association between stress level and academic grades [16]. Stress may impair memory by affecting an individual's capacity to encode and retrieve information or causing oversecretion of the stress hormone cortisol [17]. Studies have found that stress can cause students to have lower motivation, distress, exhaustion, and poorer long-term memory retention [18]. Additionally, it can impair students' sleep and self-esteem, further perpetuating poor academic performance [15].

Following academic stress, the second highest stressor was found to be relationships with peers, followed by relationships with teachers with a total of 13.6% students reporting either of these factors as a significant source of stress. Other studies have identified the significant negative impact COVID-19 has had on student's social network and interpersonal relationships internationally. In a study from Jordan, 65.6% of medical students reported worsened social relationships [19], and 86% of medical students in a US study reported decreased social interactions [20]. This was due to a variety of factors including social distancing as well as the switch to online learning [21]. The link between strength of social network and academic performance has been established in multiple studies, finding that students with a larger social circle and stronger interpersonal relationships tend to perform better. For example, a study conducted on 226 undergraduate students demonstrated a statistically significant association between the size and strength of one's social network and their academic performance [22]; this was echoed in another study from Turkey [23]. Although the results for these factors were not statistically significant, the trends identified point towards a potential link between interpersonal relationships and academic performance that can be further investigated on a larger scale.

Conversely, relationships with family members were the least reported source of stress, with only 1.8% of total survey respondents identifying it as an extremely stressful factor. This may be because many students stayed home during the pandemic and spent more time with family members as compared to before COVID-19. Another study from Saudi Arabia also found that university students reported increased time spent with family as a positive outcome of the COVID-19 pandemic [24]. Similarly, a study on medical students in Australia found that while participants reported negative impacts of COVID-19 on social connectedness, improved family relationships was a positive impact of the pandemic [25]. However, a study focusing on medical students in the US found that living at home caused increased distractibility and revealed family tensions for some students, contributing to stress and decreasing work productivity [20].

Our study had several strengths, including the use of a comprehensive and validated measurement tool -- the COVID-19 Student Stress Questionnaire. This measurement tool allowed for a targeted assessment of the various sources of stress among this specific population. Additionally, the examination of a highly relevant population of final year medical students, with their impending graduation, facilitates the efficient identification of interventions to alleviate stress and potentially improve academic performance; this may have further advantages in terms of better preparing this group for entry into the health workforce. The case-control study design was also appropriate, given the less common nature of the cases. However, there were also multiple weaknesses. Due to the retrospective nature of the study with self-reported outcomes, there may be potential selection and response bias from those who did not perform as well; they may be less likely to participate in the study or more likely to attribute their academic performance to stress. Since there was no baseline measurement prior to the pandemic for comparison, other changes such as curriculum modifications not in relation to the pandemic may also have led to stress. Finally, confounding factors such as socioeconomic status may also have impacted this association.

Stress levels, especially in relation to academics, were higher among students who failed in any subject in the final examination of the medical curriculum at our institution. These findings suggest the need to focus stress-prevention strategies on alleviating academic stress, which may in turn improve vulnerable students' academic performance [26]. Students would benefit from enhanced academic support, whether formally from institutions or informally from peers and relatives, to facilitate transitions between learning environments that pose an additional burden to students, such as the change from conventional face-to-face learning to online distance learning as implemented by numerous medical schools during COVID-19 [16]. Furthermore, higher education institutions should be proactive in screening students’ mental health to evaluate for the need of early intervention. Dedicated support programmes can be offered early to students identified as having high levels of academic stress in order to prevent the deleterious consequences of stress on future academic performance and halt the vicious cycle of stress and poor performance [13]. Stress management programmes for students would also benefit from being integrated into the formal curriculum [26]. In the new era of normal where online pedagogical methods are likely to play an integral and permanent role in medical education, innovative methods for providing academic and social support should be developed simultaneously, such as near-peer teaching programmes, online social support networks or wellness groups, and online psychotherapy and counselling [26, 27, 28].

We recognise the inherent weakness of this study. Questionnaire study is subjected to response bias - Students who underperformed in the exam may respond to evaluation questionnaires to ventilate their learning difficulties. The failure rate of the current cohort was 10% which was comparable to the previous academic years. Therefore we believe response bias is not present in this study.

This study is the first study to evaluate the impact of different stresses on academic outcome of medical students. CSSQ is a validated questionnaire to estimate the stress response on seven domains. However, CSSQ is limited by the intrinsic oversimplified design of the questionnaire. As such we believe qualitative research is needed to explore sources of stress among students to further explore different sources of stress among students and to better understand their perceptions of the impacts of such stressors on their performance. Additionally, prospective studies should be conducted to minimise response bias and better identify the impact of stressors on academic performance. Given the potential residual impacts of COVID-19 on medical education and student stress, longitudinal cohort studies can also be conducted to observe the long-term sequelae of COVID-19 related stressors and measure stress levels post-COVID-19. To increase the power of the study, a larger sample size may be recruited by including other cohorts of medical students. This could also help to demonstrate how stress levels and their precipitating factors differ across various stages of undergraduate medical education. If academic support systems and mental health services are to be enacted as previously recommended, studies should be conducted to assess target students’ needs prior to implementation to ensure that programmes are student-centered. Furthermore, research should evaluate the outcomes of such interventions, with particular focus on how they are received by students, as the success of interventions is largely dependent on how useful students themselves perceive them to be [18].

## 5 Conclusion

Academic stress during COVID-19 pandemic is associated with worse academic performance among undergraduate medical students in Hong Kong. Through identifying contextually-relevant sources of stress and delineating the mechanisms by which they impact academic performance in future research, more tailored interventions can be developed to address students’ needs in improving their mental health and consequently their academic performance.

## Declarations

### Author contribution statement

Michael Co: Conceived and designed the experiments; Performed the experiments; Analyzed and interpreted the data; Contributed reagents, materials, analysis tools or data; Wrote the paper.

Kam Sheung Poon: Conceived and designed the experiments; Analyzed and interpreted the data; Contributed reagents, materials, analysis tools or data; Wrote the paper.

Margaret Kay Ho, Alina Ashok Bharwani, Vernice Hui Yan Chan and Evelyn Hui Yi Chan: Performed the experiments; Contributed reagents, materials, analysis tools or data.

### Funding statement

This research did not receive any specific grant from funding agencies in the public, commercial, or not-for-profit sectors.

### Data availability statement

Data will be made available on request.

### Declaration of interests statement

The authors declare no conflict of interest.

### Additional information

No additional information is available for this paper.
